# Risk of septic knee following retrograde intramedullary nailing of open and closed femur fractures

**DOI:** 10.1186/1749-799X-7-7

**Published:** 2012-02-17

**Authors:** Jason J Halvorson, Marc Barnett, Ben Jackson, John P Birkedal

**Affiliations:** 1 Department of Orthopaedic Surgery, Wake Forest University Baptist Medical Center, Medical Center Blvd, Winston-Salem, NC 27103, USA; 2 Asheville Orthopaedic Associates, 11 Victoria Road, Asheville, NC 28801, USA; 3 Department of Orthopaedic Surgery, 1001 Blythe Blvd. Medical Center Plaza Charlotte, Charlotte, NC 28203, USA

## Abstract

**Background:**

One potential complication of retrograde femoral nailing in the treatment of femur fractures is the risk of septic knee. This risk theoretically increases in open fractures as a contaminated fracture site has the potential to seed the instrumentation being passed in and out of the sterile intraarticular starting point. There are few studies examining this potential complication in a relatively commonly practiced technique.

**Methods:**

All patients who received a retrograde femoral nail for femur fracture between September 1996 and November 2006 at a Level 1 trauma center were retrospectively reviewed. This yielded 143 closed fractures, 38 open fractures and 4 closed fractures with an ipsilateral traumatic knee arthrotomy. Patient follow-up records were reviewed for documentation of septic knee via operative notes, wound culture or knee aspirate data, or the administration of antibiotics for suspected septic knee.

**Results:**

No evidence of septic knee was found in the 185 fractures examined in the dataset. Utilizing the Wilson confidence interval, the rate of septic knee based on our population was no greater than 2%, with that of the open fracture group alone being 9%.

**Conclusions:**

Based on these results and review of the literature, the risk of septic knee in retrograde femoral nailing of both open and closed femoral shaft fractures appears low but potentially not insignificant.

**Funding:**

There was no outside source of funding from either industry or other organization for this study.

## Introduction

Fractures of the femur are a common injury encountered by orthopaedic surgeons. Intramedullary nailing has become the gold standard for treatment of shaft fractures and has been routinely employed since the 1970's[1-4]. Traditionally, femoral nails have been inserted in an antegrade fashion with a start point at the piriformis fossa or the greater trochanter. Retrograde nails utilizing an intra-articular insertion point at the knee with a retrograde trajectory for femoral nailing has been described [5] and has gained great popularity in recent years. As the indications for retrograde nailing continue to evolve [6-15], several publications have reported comparable outcomes following retrograde or antegrade femoral nailing. The retrograde technique confers potentially unique complications as compared with the antegrade technique. One area of concern involves infection of the knee joint [8,9,16]. There are many proposed mechanisms for development of a septic knee with the retrograde femoral nail technique. The repeated passage of instrumentation (reamers) through the intraarticular start point at the knee may potentially contaminate the sterile joint space. Similarly, in open fractures, passing reamers through the "contaminated" open fracture and out through the knee joint is thought to potentially enhance the risk of septic arthritis. After closure, the nail provides a hypothetical conduit for bacteria from the fracture site to enter the knee joint. One author has specifically stated that "severe grade III open fractures probably should not be treated with a retrograde nail," [9] based on the occurrence of a late septic knee in a patient treated with this technique.

The specific aim of the current study was to review the clinical outcomes of all retrograde femoral nails performed at a Level I trauma center over a 10 year span with a focus on the incidence of septic knee in patients with either open or closed fractures. Our hypothesis was that patients undergoing retrograde femoral nailing are not at a significantly increased risk of knee sepsis following treatment for open or closed femur fractures.

## Methods

After internal Institutional Review Board approval, and in compliance with the Helsinki Declaration, a list of all patients undergoing operative treatment for femoral shaft or supracondylar femur fracture between September 1996 and November 2006 at a Level 1 trauma center was generated from CPT codes. This generated a list of 1,163 fractures for potential analysis. Operative notes from these patients were reviewed to identify all patients with a retrograde nail placed via the standard described technique using an intraarticular starting point. There were no original exclusion criteria for those included into the database. A total of 309 patients with 352 fractures were originally identified as meeting inclusion criteria.

Patients were then divided into two groups: those with greater and those with fewer than six months of orthopaedic follow-up. Attempts were made to contact patients with less than 6 months orthopaedic follow-up via phone using the hospital's database of patient demographic information, including phone numbers and listed emergency contacts. If the hospital demographic information was incorrect, a commercial website, peoplefinders.com ™, was accessed for additional patient contact information. Patients who were successfully contacted were asked to complete a phone questionnaire about their post-operative course, including the need for knee aspiration, antibiotics, cultures, or open/closed procedures for irrigation/debridement of the knee. Contact was successfully made with an additional 14 patients (14 fractures); 11 within the closed fracture group and 3 within the open fracture group. Most patients who could not be contacted had an incorrect or disconnected phone number.

Excluding those patients without documented orthopaedic follow-up of greater than six months, 245 fractures out of the original 352 fractures remained. Open and closed fractures were analyzed separately to determine the risk of septic knee within each sub-population of patients who received a retrograde femoral nail. For statistical analysis, all patients with bilateral fractures were excluded as to prevent introduction of bias. This removed 30 total patients from analysis, leaving 185 patients for final analysis (143 closed fractures, 42 open fractures). There was a sub-population identified within the open fracture group which sustained a traumatic knee arthrotomy with associated closed femur fracture. Four patients fit this criterion and were excluded, bringing the number of patients with open femur fracture to 38. Three fractures were pathologic in nature. Eleven retrograde nails were used for peri-prosthetic knee or hip fractures. One hundred seventeen patients were considered poly-trauma.

A retrospective chart review of patients with the remaining 245 fractures was performed to further characterize open vs closed injury, the grade of open injury when applicable, and complications. All open fractures were treated with emergency department bedside irrigation and debridement and antibiotics. There is no standardized protocol for antibiotic type and for how long those antibiotics are continued post-operatively for open fractures within our institution as this is instructor dependent. Therefore, the role of type, duration, and affect of antibiotic was impossible to examine, document, and control. They were then taken to the operating room as soon as possible pending trauma clearance and operating room availability. The only reason patients with open fractures were not taken to the operating room in an urgent or emergent fashion was secondary to life threatening traumatic or neurosurgical injuries which precluded orthopaedic intervention. In these cases, antibiotics were continued and the patient was taken to the operating room for formal irrigation and debridement when medically stable. Post-operative medical records were reviewed for documentation of infections, knee wounds, knee cultures/aspirates, and signs or symptoms of knee infection. A definition of septic knee was pre-defined as the identification of positive cultures from a knee aspirate, intraoperative irrigation and debridement, or any clinical signs and symptoms of deep knee infection (as opposed to superficial wound infection).

Time to union, rates of non-union, diagnosis of osteomyelitis, and other complications were not tabulated in our analysis as they were out of the scope of our hypothesis with regard to the documented occurrence of septic knee.

A confidence interval was chosen for statistical analysis of the data. However, given the low occurrence rate of septic knee within our population, the author's utilized Wilson's Confidence Interval for data analysis in hopes to more accurately capture the true incidence of septic knee.

Demographic data from the fracture database is enclosed in Table 1. Overall follow-up rate of patients with greater than 6 month documented orthopaedic evaluation was 70%. Open injuries were classified as defined by the Gustillo-Anderson classification system [17]. Of the open injuries, 7 were Gustillo-Anderson type I, 17 were type II, 7 were type III, and 7 were simply reported as gunshot wounds (Table 2).

**Table 1:** Demographic Data

	**Combined Open/Closed Fractures**	**Closed Fractures**	**Open Fractures**	**Arthrotomy with Closed Femur Fracture**
Total Patients with at least 6 month follow-up	185	143	38	4
Average age yrs (range)	38 (14-91)	40 (14-91)	33 (17-69)	28 (17-49)
Number Males/Females	94/91	84/107	30/8	0\4
Left/Right Knee	105/80	63/80	23/15	3\1
Average follow-up, months (range)	29 (6-116)	28 (6-114)	30 (6-116)	37 (6-116)

**Table 2:** Break-down by Gustillo-Anderson Classification of Open Fractures

Fracture Grade (Gustillo-Anderson)	Number
GI	7
GII	17
GIII	7
Gunshot	7
Traumatic Arthrotomy with Ipsilateral Femur Fracture	4

## Results

Nine patients were noted to have documented infections requiring return trip to the operating room: four within the closed fracture cohort and five within the open fracture cohort (overall rate 3%). Five of the nine patients were documented to have an infected nonunion, three of which were within the open fracture cohort. Two patients were found to have osteomyelitis with fracture union (one in each group). Two patients were found to have wound infections requiring irrigation and debridement (one in each group) without evidence of deep infection. There was no documentation within the medical records of an occurrence of septic knee; specifically, there were no reports of positive knee aspirate cultures, surgical procedures for knee sepsis, exchange nailing for suspected knee sepsis, or antibiotics (intravenous or oral) prescribed for suspected septic knee infection. The calculated Wilson confidence interval for septic knee after retrograde femoral nailing for each sample size and population can be found in Table 3. Using our sample size of 185 patients, the Wilson confidence interval of pyarthrosis following retrograde femoral nailing (either open or closed fracture) was found to be 0% - 2% using a 95% confidence interval. The Wilson confidence interval for septic knee following closed retrograde nailing using our sample size of 143 fractures was 0% - 2.6% with a 95% confidence interval. When looking at the open fracture group alone, the Wilson confidence interval for septic knee following a retrograde nail was 0% - 9% with a 95% confidence interval. (Table 3)

**Table 3:** Incidence of septic knee utilizing the Wilson Confidence Interval (95% Confidence Interval) for all patients treated with retrograde intramedullary nail and retrograde nail treatment separated into those patients with open and closed femur fractures

	Patients	Wilson Confidence Interval For Septic Knee
Total	185	2%
Open	38	9%
Closed	143	2.60%

## Discussion

In the review of our data, there was no record of a septic knee within the medical record documentation at our Level I trauma center in a 10 year span when retrograde femoral nailing was used for treatment of both open and closed femoral fractures. We were able to follow 70% of patients greater than 6 months who received a retrograde femoral nail within the 10 year span. Utilizing the Wilson confidence interval, the overall rate of septic knee when using the retrograde femoral nail technique for femur fractures at our institution would be expected to be no greater than 2% with a 95% confidence interval. Isolating the open femur fracture group, the Wilson confidence interval for finding a pyarthrosis following retrograde nail rises to 9% with a 95% confidence interval which is not insignificant given the potential devastating consequences of this complication. The small sample size limits definitive conclusions regarding the safety of retrograde nails for open femur fractures. While, in practice, we routinely use retrograde femoral nails in association with open femur fractures, our data at least suggest the possibility that knee pyarthrosis is a concern following this technique.

In addition, four patients sustained a traumatic arthrotomy at the time of injury associated with ipsilateral closed femur fracture. An advantage of a retrograde approach in this condition is that the surgical exposure in these cases is essentially already present allowing the placement of the retrograde nail via the traumatic arthrotomy. The concern in utilizing a retrograde nail in this situation is similar to the situation of an open fracture in that a potentially contaminated wound may contaminate the inserted hardware. The low number of patients in this group precludes a definitive conclusion about the risk of septic knee when a traumatic arthrotomy is present. However, none of these patients had report of a septic knee.

The rare rate of septic knee within our cohort is consistent with the current literature. In the one systematic review of retrograde femoral nails which included 914 patients in 24 journal articles, only one septic knee was identified for an overall incidence of 0.18% [10]. No breakdown of statistics was performed comparing open vs closed fractures and rates of infection. Other reported rates of septic knee following retrograde nailing of femur fractures is low [9,16]. Finally, a recent analysis by O'Toole et al. demonstrated overall rate of septic knee in open femur fractures at 1.1% in 90 patients [18]. While their reported rate of septic knee is low, they admit to being underpowered despite their multi-center study, demonstrating the magnitude of patients necessary and difficulty in soundly answering this question.

Weaknesses to this study include its retrospective nature and the bias this inherently creates. Also, low patient numbers preclude definitive recommendations as to the safety regarding the occurrence of septic knee following this retrograde nailing for femur fractures. In addition, the self-imposed use not including patients with less than 6 month follow-up excluded a number of patients. Patients with less than six month follow-up were arbitrarily excluded for their relatively short-term follow-up and potential lack of information regarding complications of the procedure. Including these patients would potentially offer false negatives into the final statistical analysis. The majority of patients with less than 6 month follow-up were evaluated at clinic visits between 1-6 months. Review of these 81 medical records provided no evidence of septic knee by the definitions used above (data not included).

With use of the Wilson confidence interval, we were able to demonstrate a low rate of septic knee with global use of the retrograde nailing technique when used for femur fractures, regardless of being open or closed fractures. However, the risk of septic knee in a closed fracture and sterile operating room environment with sterile tools confers little increased risk of septic knee. The concern comes with a contaminated open fracture site with reamers passing in and out of a 'sterile' intra-articular space. The trend from our data certainly appears to suggest a low incidence of septic knee when using retrograde nails for both open and closed fractures. However, we cannot definitively state with absolute statistical significance that the risk of septic knee for either open or closed femur fracture treated via a retrograde technique is negligible. While this study should offer the practicing orthopaedic surgeon confidence that the overall risk of septic knee with this technique for treating either open or closed femur fractures is low, further study with larger numbers is necessary in order to definitively confirm the exact incidence and rate of this complication.

## Competing interests

The authors declare that they have no competing interests.

## Authors' contributions

All authors were involved with manuscript idea, preparation, data collection/analysis, statistical analysis, and writing/revision of manuscript. All authors have approved the above manuscript.
